# Understanding LiOH Chemistry in a Ruthenium‐Catalyzed Li–O_2_ Battery

**DOI:** 10.1002/anie.201709886

**Published:** 2017-11-21

**Authors:** Tao Liu, Zigeng Liu, Gunwoo Kim, James T. Frith, Nuria Garcia‐Araez, Clare P. Grey

**Affiliations:** ^1^ Department of Chemistry University of Cambridge Lensfield Road Cambridge CB2 1EW UK; ^2^ Department of Chemistry University of Southampton Highfield Campus Southampton SO17 1BJ UK

**Keywords:** dimethyl sulfone, Li–O_2_ batteries, LiOH, oxygen reduction/evolution, ruthenium catalysis

## Abstract

Non‐aqueous Li–O_2_ batteries are promising for next‐generation energy storage. New battery chemistries based on LiOH, rather than Li_2_O_2_, have been recently reported in systems with added water, one using a soluble additive LiI and the other using solid Ru catalysts. Here, the focus is on the mechanism of Ru‐catalyzed LiOH chemistry. Using nuclear magnetic resonance, operando electrochemical pressure measurements, and mass spectrometry, it is shown that on discharging LiOH forms via a 4 e^−^ oxygen reduction reaction, the H in LiOH coming solely from added H_2_O and the O from both O_2_ and H_2_O. On charging, quantitative LiOH oxidation occurs at 3.1 V, with O being trapped in a form of dimethyl sulfone in the electrolyte. Compared to Li_2_O_2_, LiOH formation over Ru incurs few side reactions, a critical advantage for developing a long‐lived battery. An optimized metal‐catalyst–electrolyte couple needs to be sought that aids LiOH oxidation and is stable towards attack by hydroxyl radicals.

Non‐aqueous Li–O_2_ batteries possess a high theoretical energy density that is 10 times higher than that of the current lithium ion batteries.^1^ There have been considerable efforts from academia and industry in the past decade to understand and realize this battery system. Despite much research investment, significant challenges remain. One of the most fundamental problems concerns the side reactions that occur during cell cycling.^2^ During battery discharge, O_2_ is reduced to form Li_2_O_2_ via an intermediate LiO_2_;^3^ on charging Li_2_O_2_ decomposes releasing O_2_.3a, 4 Both the superoxide and peroxide (either as solvated ions or solid phases) are highly reactive and their formation/decomposition can cause electrolyte and electrode decomposition,2d, 5 especially in the presence of high overpotentials. As a result, many groups have been searching for new Li–O_2_ battery chemistries.^6^, ^7^, ^8^

Recently, LiOH has been identified as the major discharge product in a few of Li–O_2_ battery systems and reversible electrochemical performance has been shown.^7^, ^8^ One case published by some of the authors,^7^ concerns the use of a soluble catalyst LiI, which catalyzes the LiOH formation with its H source solely coming from added H_2_O in the electrolyte; a subsequent study^9^ confirmed the proposed 4 e^−^ oxygen reduction reaction (ORR) on discharging. It was also shown on charging that the LiOH can be removed with the aid of LiI_3_ at around 3.1 V.^7^ The other case employs a Ru‐based solid catalyst in a water‐added dimethyl sulfoxide (DMSO) or tetraglyme electrolyte.^8^ Ru was proposed to catalyze LiOH formation and decomposition in a tetraglyme electrolyte with 4600 ppm water. In the DMSO case it was suggested that at low water contents (ca. 150 ppm), a mixture of Li_2_O_2_ and LiOH was formed on discharge, and that on charging, Li_2_O_2_ was first converted to LiOH, the latter then being decomposed by Ru catalysts at voltages of as low as about 3.2 V. At higher water contents (ca. 250 ppm), LiOH formation appeared to be dominant on discharge.^10^ It is clear that understanding the formation and decomposition of LiOH is not only critical in helping realize a LiOH‐based Li–O_2_ battery, but fundamental insight into LiOH based chemistries may also aid in the development of Li_2_O_2_‐based batteries that operate utilizing air (or moist oxygen), where LiOH inevitably forms.

Herein we develop a mechanistic understanding of the Ru‐catalyzed oxygen chemistry. Using quantitative nuclear magnetic resonance and operando electrochemical pressure and mass spectrometry measurements, we show that on discharging, a total of 4 electrons per O_2_ is involved in LiOH formation, this process incurring fewer side reactions compared to Li_2_O_2_. On charging, the LiOH is quantitatively removed at 3.1 V, with the oxygen being trapped in the form of soluble dimethyl sulfone in the electrolyte.

The preparation of the Ru/SP (SP; Super P) carbon electrode is described in the Supporting Information. Microscopy and diffraction experiments show that Ru crystals of less than 5 nm are well dispersed on the SP carbon substrate (Supporting Information, Figure S1). Figure 1 A shows typical electrochemical profiles of Li–O_2_ batteries prepared using Ru/SP electrodes with various concentrations of added water in a 1 m LiTFSI/DMSO (lithium bis(trifluoromethane) sulfonimide in dimethyl sulfoxide) electrolyte. In the nominally anhydrous case, discharge and charge plateaus are observed at 2.5 and 3.5 V, respectively, where an electrochemical process involving two‐electrons per oxygen molecule and Li_2_O_2_ formation dominates on discharging (Supporting Information, Figure S2). As the water content increases, it is clear (Figure 1 A) that the voltage gaps between discharge and charge reduce considerably. With 50 000 ppm water, the cell discharges at 2.85 V and charges at 3.1 V, although further increasing the water content then widens the voltage gaps (Supporting Information, Figure S3). Figure 1 B shows the electrochemistry of cells made using various metal catalysts and 1 m LiTFSI/DMSO electrolyte with 4000 ppm water. Although the discharge voltages are all similar, and close to 2.7 V, clear differences are observed on charging, where Ir, Pd, Pt all show charging voltages beyond 3.5 V while for Ru it is only 3.2 V, demonstrating the crucial role of metal catalysis on the charging process. Examining the discharged Ru/SP electrodes, two distinct morphologies were observed for the discharge product (Figure 1 C,D): at lower water contents (for example, 4000 ppm), cone‐shaped particles dominate whereas at higher water contents (for example, 50 000 ppm), flower‐like large agglomerates formed; these morphologies were observed before for LiOH crystals.^7^ Indeed, both X‐ray diffraction (XRD) and Raman measurements suggest that in the current Ru‐based system, LiOH is the only discharge product observed with 4000‐50 000 ppm added water; no evidence of other chemical species commonly observed in Li–O_2_ batteries, such as Li_2_O_2_, Li_2_CO_3_, and HCOOLi, is seen by XRD and Raman spectroscopy (Figure 1 E,F). Ir and Pd catalysts also invariably lead to LiOH formation (Supporting Information, Figure S4).

**Figure 1:**
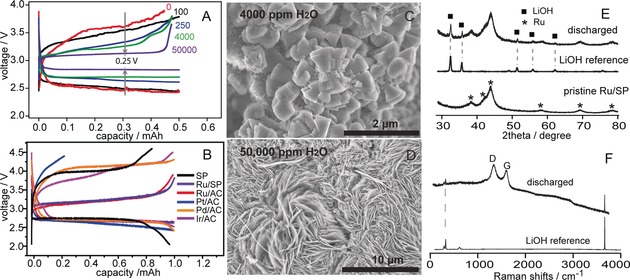
A),B) Electrochemical profiles of Li–O_2_ cells with A) different water contents (in ppm) in a 1 m LiTFSI/DMSO electrolyte and B) using different metal catalysts (AC=activated carbon, SP=super P). C)–F) Characterization of discharged electrodes by SEM (C,D), XRD (E), and Raman spectroscopy (F). All cells in (A) use Ru/SP electrodes; All cells in (B) contain a water content in the electrolyte of 4000 ppm. All cells were cycled at a current of 50 μA (0.1 mA cm^−2^). The discharged electrodes measured in XRD (E) and Raman (F) are both prepared using the electrolyte with 4000 ppm water and Ru/SP electrodes.

To demonstrate that at water levels beyond 4000 ppm, LiOH is formed from O_2_ reduction rather than from electrolyte decomposition, we performed NMR experiments with isotopically labeled H (D_2_O) and O (H_2_^17^O, ^17^O_2_) (Figure 2 A–C). When natural abundance DMSO and H_2_O were used, a dominant ^1^H NMR resonance at −1.5 ppm attributed to LiOH was observed (Figure 2 A).^7^, ^11^ Using D_2_O, we found a distinct first‐order quadrupolar‐broadened line shape for LiOD in the ^2^H NMR spectrum (Figure 2 B);^7^ when deuterated [D_6_]DMSO and H_2_O were used, hardly any LiOD signal was seen (Figure 2 B) and LiOH was the prevailing product (Figure 2 A). The proton in LiOH thus comes overwhelmingly from the added water in the DMSO electrolyte. Next, we enriched either gaseous O_2_ or H_2_O with ^17^O to verify the O source in LiOH. In both cases, the resulting ^17^O NMR spectra (Figure 2 C) revealed a resonance at around −50 ppm with a characteristic second‐order quadrupolar line shape, which is ascribed to LiOH.^11^ It is thus clear that both oxygen atoms in O_2_ and H_2_O contribute to the formation of LiOH, consistent with a four‐electron ORR.

**Figure 2:**
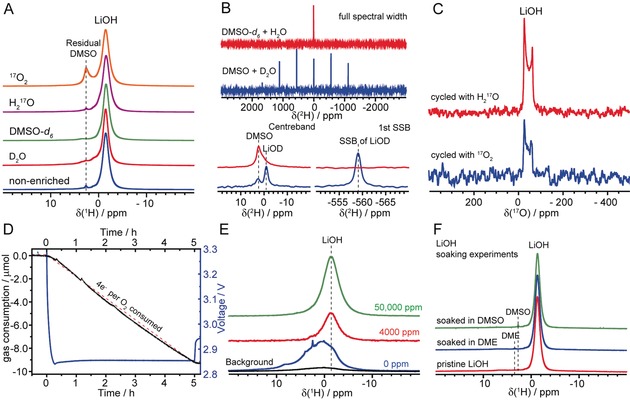
A)–C) ^1^H (A), ^2^H (B), and ^17^O (C) solid‐state NMR spectra of discharged Ru/SP electrodes, prepared from Li–O_2_ cells with 1 m LiTFSI/DMSO electrolyte with 4000 ppm water. The ^1^H NMR spectra (A) show that all samples, independent of the nature of isotope enrichment (as labeled), give rise to a dominant resonance at −1.5 ppm corresponding to LiOH; the small resonance at 2.5 ppm is due to residual DMSO. The ^2^H NMR spectra confirm that water is the proton source for LiOH formation. Note that the ^1^H NMR experiments in (A) are not quantitative, and the LiOH detected in the case with added D_2_O in the DMSO‐based electrolyte is likely due to H_2_O impurities from D_2_O. D) Operando pressure measurement of a Ru‐catalyzed cell with 50 000 ppm water and E) quantitative ^1^H NMR spectra of first discharged electrodes prepared from Li–O_2_ cells using 1 m LiTFSI/DMSO electrolyte with 0, 4000, and 50 000 ppm water contents. 10 μmol O_2_ consumption corresponds to 27.7 mbar pressure drop measured for 1 mAh capacity (200 μA, 5 hours). F) ^1^H NMR evaluating the long‐term stability of LiOH in DMSO and DME solvents by comparing LiOH powder with those after being soaked in DMSO and DME solvents for a month. Apart from the residual DMSO or DME solvent, no additional signals are observed, the soaked LiOH powder remaining chemically unchanged.

Operando pressure measurements show that the recorded pressure matches well with the trend line expected for 4 e^−^ per O_2_ (Figure 2 D), providing additional verification of this mechanism. Therefore, we propose an overall discharge reaction as follows: O_2_+4 e^−^+4 Li^+^+2 H_2_O→4 LiOH (1). Up to four electrons can be stored per O_2_ molecule, the theoretical capacity of the battery operating via Reaction (1) being 1117 mAh/g_LiOH_, comparable to Li_2_O_2_ (1168 mAh/g${}_{\mathrm{Li}{}_{2}O{}_{2}}$ ). To examine the role of Ru in LiOH formation further, we discharged a SP electrode in a 1 m LiTFSI/DMSO electrolyte with 4000 ppm water. XRD and SEM show that the discharge leads to mainly Li_2_O_2_ formation with an e^−^/O_2_ ratio of 2.2 (Supporting Information, Figure S5), whereas discharging Ru/SP in the same electrolyte forms only LiOH. This contrasting behavior suggests that in the absence of Ru, the reaction between H_2_O and Li_2_O_2_, 2 Li_2_O_2_+2 H_2_O→4 LiOH+O_2_ (2), is slow, even though it is thermodynamically favorable (Δ*G*°=−149.3 kJ mol^−1^) but Ru clearly promoted the LiOH formation. By exposing a Ru/SP electrode discharged in a nominally dry electrolyte (where Li_2_O_2_ is the main product) to the 4000 ppm water‐added electrolyte, XRD (Supporting Information, Figure S5) shows that all the Li_2_O_2_ was converted into LiOH in the presence of Ru after 10 h (same time period as used for the galvanostatic discharge in the SP cell); this indicates that Ru can catalyze reaction (2) given above. It is likely that the electrochemical formation of LiOH in the Ru/SP system proceeds via first Li_2_O_2_ generation (O_2_+2 e^−^+2 Li^+^→Li_2_O_2_) and then Ru catalyzes the chemical reaction of Li_2_O_2_ with H_2_O to eventually form LiOH (Reaction 2); overall the reaction is O_2_+4 e^−^+4 Li^+^+2 H_2_O→4 LiOH. This observation also suggests that water present must be important to solubilize Li_2_O_2_, LiOH, and derived species and facilitate the solid‐solid phase conversion (from Li_2_O_2_ to LiOH).

Importantly, the LiOH formation during discharge involves few parasitic reactions. Quantitative ^1^H solid‐state NMR spectra (Figure 2 E) comparing the discharged electrodes generated from an anhydrous electrolyte versus those with 4000 and 50 000 ppm added water shows that the Li_2_O_2_ chemistry (at the anhydrous conditions) clearly generated Li formate, acetate, methoxide side reaction products (signified by the resonances at 0–10 ppm),^7^, ^11^ whereas only a single resonance at −1.5 ppm was seen in the LiOH chemistry; similar results were observed with the other metal catalysts (Supporting Information, Figure S3). Furthermore, we found that soaking LiOH in dimethoxyethane (DME) and DMSO for a month showed no change in its solid state NMR spectra (Figure 2 F), indicating that LiOH is chemically inert in these solvents. ^1^H and ^13^C solution NMR measurements of electrolytes extracted after electrochemical discharge and from a slurry of LiOH and electrolyte held under O_2_ for 30 days also show that hardly any soluble side‐reaction products are formed (Supporting Information, Figure S6).

Now moving to battery charging, this process was characterized by ex‐situ NMR and XRD measurements of electrodes after multiple cycles, as presented in Figure 3 A–C. Both these series of measurements consistently show that quantitative LiOH formation on discharging and LiOH removal (even at 3.1 V) on charging are the prevailing processes during cell cycling. Hardly any residual solid, side‐reaction products accumulate in the electrode over extended cycles. Typically, the cells can cycle over 100 cycles at 1 mAh cm^−2^ (0.5 mAh or 1250 mAh/g_Ru+C_ per cycle), with consistent electrochemical profiles (Supporting Information, Figure S7). Although the ex situ tests supported a highly reversible O_2_ electrochemistry, operando electrochemical pressure and mass spectrometry experiments suggested otherwise: very little gas was evolved on charging (Figure 3 D,E) and the pressure of cell continues to drop over extended cycles (Figure 2 F); these observations imply that oxygen must be trapped and accumulate after charging in the cell, likely in the electrolyte.

**Figure 3:**
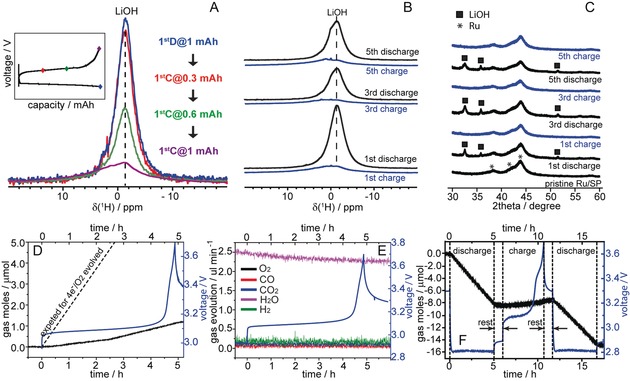
A)–C) Quantitative ^1^H (A,B) and ex situ XRD measurements (C) of cycled Ru/SP electrodes prepared using 1 m LiTFSI/DMSO electrolytes with 50 000 ppm water; D)–F) operando electrochemical pressure (D,F) and mass spectrometry (E) measurements of a Ru‐catalyzed cell with 50 000 (D,E) and 4000 ppm (F) water. Batteries terminated both at different state of charge (A) and fully charged following different discharge–charge cycles all show quantitative electrochemical removal of LiOH. Little O_2_ evolution is seen during charging (D, E) and the cell pressure continues to drop over extended cycles (F). 10 μmol O_2_ in (D) and (F) corresponds to 27.7 mbar pressure change measured for 1 mAh capacity (200 μA, 5 h).

Further solution‐NMR measurements were performed on electrolyte samples prepared from several charged cells extracted following different cycle numbers, where ^17^O enriched H_2_O (H_2_^17^O) was used in the electrolyte. Figure 4 shows the ^1^H (A), ^13^C (B), and ^17^O (C), and ^1^H–^13^C heteronuclear single quantum correlation (D) solution NMR spectra of the cycled electrolytes. New peaks at 2.99 ppm (^1^H), 42.6 ppm (^13^C), and 169 ppm (^17^O) appeared and progressively intensified with increased cycle number; these resonances consistently point towards the formation of dimethyl sulfone (DMSO_2_), its identity being further corroborated in the ^1^H–^13^C correlation spectrum. Of note, the ^17^O signal of DMSO_2_ is even stronger than the large amount of natural abundance (NA) DMSO used in the solution NMR experiment, suggesting that DMSO_2_ is likely to be ^17^O‐enriched. Its growth in intensity is accompanied by the decrease of H_2_^17^O, indicating that some ^17^O from H_2_^17^O ended up in DMSO_2_ owing to isotope scrambling in the charging process. Given that LiOH is quantitatively formed and then removed on charge (Figure 3), we propose that the charging reaction is initiated by electrochemical LiOH oxidation to produce hydroxyl radicals, which then chemically react with DMSO to form DMSO_2_: LiOH→Li^+^+e^−^+^.^OH (3); DMSO+2 ^.^OH →DMSO_2_+H_2_O (4). The overall reaction thus is: 2 DMSO+4 LiOH→2 DMSO_2_+2 H_2_O+4 e^−^+4 Li^+^ (5). It is seen that the same number of electrons is involved in discharge (Reaction 1) and charge (Reaction 5), with one O reacting per two electrons (as expected for O_2_ evolution reaction, OER). The electrochemical process, Reaction (3), sets the voltage observed on charging, rather than the overall Reaction (5). Surface adsorbed hydroxyl species are generally considered to be the first reaction intermediates on many OER metal catalysts in aqueous media.^12^ The added water in the current electrolyte could promote LiOH dissolution, and thus facilitate the access of Ru surfaces to soluble LiOH species resulting in the formation of surface hydroxyl species. Once the radical is formed on charging, it is consumed by reacting with DMSO to form DMSO_2_ and thus the battery can be continuously charged at a low voltage until all solid LiOH products are removed (see further discussion in the Supporting Information, Figure S8). The resulting DMSO_2_ is soluble in the DMSO electrolyte and will not immediately impede ion diffusion or interfacial electron transfer as other insoluble by‐products would do, which is perhaps why this side reaction does not rapidly lead to battery failure.

**Figure 4:**
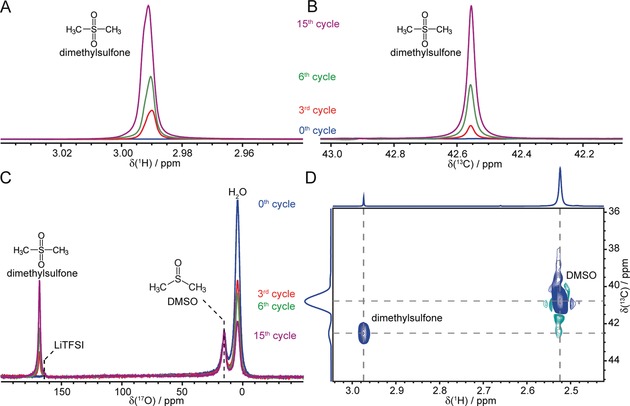
A)–C) ^1^H (A), ^13^C (B), and ^17^O (C) and ^1^H–^13^C heteronuclear single quantum correlation (D) solution NMR spectra of cycled 1 m LiTFSI/DMSO electrolyte with 45 000 ppm ^17^O enriched water from Ru‐catalyzed Li–O_2_ batteries. New resonances at 2.99 ppm (^1^H), 42.55 ppm (^13^C) and 169 ppm (^17^O) signify the formation of DMSO_2_. The heteronuclear correlation experiment was performed on a charged electrolyte at the end of the 6th cycle. The cross‐peak at (2.99 ppm ^1^H −42.55 ppm ^13^C) further supports DMSO_2_ formation; the other cross‐peak at (2.54 ppm ^1^H −41.0 ppm ^13^C) is due to DMSO.

In summary, we have shown that with added water (beyond 4000 ppm) in the electrolyte, the Ru‐catalyzed battery chemistry changes from Li_2_O_2_ to LiOH formation, similar reactions being seen for several other metal catalysts. The cell discharge reaction consumes four electrons per reduced O_2_ molecule. This LiOH formation process involves very few side reactions and LiOH itself is much more stable in organic solvents than Li_2_O_2_; these are the fundamental prerequisite for a long‐lived Li–O_2_ battery. On charging, the Ru quantitatively catalyzes LiOH removal via DMSO_2_ formation rather than O_2_ evolution. We propose that DMSO_2_ forms by the reaction of hydroxyl radicals with DMSO, the former being generated on Ru catalyst surfaces. This work highlights the advantage of using metal catalysts to catalyze a 4 e^−^ ORR with very few side reactions, and also the unique role of a metal catalyst in promoting LiOH formation versus electrolyte decomposition. An optimized catalyst–electrolyte couple needs to be sought for to satisfy both activity towards LiOH oxidation and stability against electrolyte decomposition on charging. This work provides a series of key mechanistic insights into the Ru‐catalyzed Li–O_2_ battery in the presence of water, which will aid the design of catalyst and electrolyte systems that can be used in more practical batteries.

## Conflict of interest

The authors declare no conflict of interest.

## Supporting information

As a service to our authors and readers, this journal provides supporting information supplied by the authors. Such materials are peer reviewed and may be re‐organized for online delivery, but are not copy‐edited or typeset. Technical support issues arising from supporting information (other than missing files) should be addressed to the authors.

Supplementary
