# Supplementary material for: Understanding LiOH Chemistry in a Ruthenium‐Catalyzed Li–O2 Battery
Source: Angew Chem Int Ed Engl. 2017 Nov 21;56(50):16057–62. doi: 10.1002/anie.201709886 (PMC6033020; doi:10.1002/anie.201709886)
Supplement: Supplementary file 1 — Supplementary [file ANIE-56-16057-s001.pdf]

Angewandte Chemie
Eine Zeitschrift der Gesellschaft Deutscher Chemiker

Supporting Information

Understanding LiOH Chemistry in a Ruthenium-Catalyzed Li–O_2 Battery

*Tao Liu, Zigeng Liu, Gunwoo Kim, James T. Frith, Nuria Garcia-Araez, and Clare P. Grey**

anie_201709886_sm_miscellaneous_information.pdf

WILEY-VCH

Contents:

Page 2: Experimental details

Page 4: Characterization of the Ru/SP pristine electrode by SEM, TEM and TGA

Page 5: Characterizing the discharging process using a nominally dry electrolyte

Page 6: Electrochemistry of the Ru-catalyzed battery with higher water contents (> 5%)

Page 7: ^{1}H and ^{7}Li solid state NMR characterization of the discharged electrodes with other metal catalysts

Page 8: The role of Ru on the catalysis of LiOH formation

Page 10: ^{1}H and ^{13}C solution NMR characterization of the electrolyte after discharge, charge and soaking with LiOH under O_2

Page 12: Long-term cycling of the Ru-catalyzed Li-O_2 battery under conditions of LiOH formation

Page 12: Further discussion on the LiOH oxidation potential with different water contents

Page 14: Author contributions

Experimental Details

Materials

A typical synthesis for Super P (SP) carbon (Timcal) supported Ru catalysts is as follows: 232 mg of $RuCl_3 \bullet xH_2O$ (Sigma Aldrich) was dissolved in deionized water (120 ml) and then 300 mg of super P carbon was added to the solution, stirred and impregnated for half an hour. The resulting solution was subsequently freeze-dried and reduced in a 5% H_2/Ar mixture atmosphere at 230°C for 1.5 hours. Dimethyl sulfoxide (Sigma Aldrich, 99%) solvent was refluxed with CaH_2 prior to fractional distillation under vacuum, and then stored over 4 Å molecular sieves. The final water content of the solvent was measured to be lower than 10 ppm by Karl Fischer titration (Metrohm 899). Lithium bis(trifluoromethyl) sulfonylimide (LiTFSI) (3M Fluorad^{TM}, HQ115) was dried at 160°C in vacuo for 12 hours before being used to prepare the 1 M LiTFSI/DMSO electrolyte. Catalysts of Ru (10%), Pd (10%), Ir (1%) and Pt (10%) supported on activated carbon (AC) were all purchased from Sigma Aldrich.

Battery assemblies and electrochemical tests

Mesoporous Super P carbon electrodes were prepared from a mixture of 80% Ru/SP and 20% PTFE (polytetrafluoroethylene) binder. The mixture was first mixed by stirring it in ethanol at 70°C and the resulting Ru/SP/PTFE mixture (lump) was kneaded and rolled into a thin film. The film was cut into electrodes of around 0.5 cm^2 and dried in vacuo at 120°C for 12 hours before using them to make batteries. The mass loading of the electrode is around 1 mg/cm^2.

All Li-O_2 cells investigated in this work are based on a Swagelok design. They were assembled by stacking a disc of lithium iron phosphate (around 40 mg total mass of LFP and PTFE binder), one piece of borosilicate glass fiber separator (Whatman) and a Ru/SP/PTFE positive electrode sandwiched between 2 stainless meshes. The electrolyte used in this study is 1 M LiTFSI/DMSO with various added water contents based on weight percentage (ppm). LFP was used as the counter electrode because of the high water contents used in the electrolyte and the instability of DMSO against Li metal. Typically, the total electrolyte volume used for a battery is 100-150 µl. The electrochemical measurements (Galvanostatic discharge/charge) were conducted using a Biologic VMP. All potentials are referenced against Li/Li^+.

Materials and battery characterization

For all batteries, the characterization of electrodes involved first disassembling the cell, rinsing the O_2 position electrode twice in dry acetonitrile (< 1 ppm H_2O), each time with 2 ml for 30 minutes. The rinsed electrodes were then dried in vacuo overnight for further characterization.

X-ray diffraction (XRD) measurements were performed using a Panalytical Empyrean operated in a reflection mode, with Cu K α1 radiation (λ=1.5406 Å). The cycled electrode was sandwiched between two Kapton polyimide films in an air tight sample holder. Scanning electron microscopy (SEM) images were recorded with a Hitachi S-5500 in lens field emission electron microscope. Transmission electron microscopy (TEM) images were recorded with a JOEL JEM 3010 microscope at 250 kV. The electrode samples were hermetically sealed during transfer to the microscope. Once the seal was opened, the electrode sample was loaded into the high vacuum chambers within 20 seconds. Raman spectra (LabRAM Horiba) were acquired using a 633 nm laser (5 seconds accumulation, averaged over 5 scans); the cycled electrodes were placed in an air tight sample holder with a quartz window.

All solid-state NMR spectra were acquired on a 16.4 T Bruker Avance III spectrometer using either 1.3 mm HX (Fig2A-C and F, and Fig. S3) or 3.2mm HXY (Fig. 2E, and Fig. 3A-B) probeheads. A rotor synchronized Hahn-echo pulse sequence was used to acquire ^1H, ^2H, ^7Li and ^{17}O magic angle spinning (MAS) spectra with a spinning speed of 55-60 kHz, with a recycle delay of 20, 3, 20, and 3 s (for ^1H, ^2H, ^7Li, and ^{17}O, respectively), and an RF field strength of 125 - 170 kHz. The ^1H quantification experiments (Fig. 2E, and Fig3A-B) were performed with a 20 kHz MAS, with a recycle delay of 150 s. The ^2H and ^{17}O NMR spectra were acquired with low power two-pulse phase-modulated (TPPM) heteronuclear decoupling scheme at an RF field amplitude of 12–15 kHz. ^1H, ^2H, ^7Li, and ^{17}O shifts were externally referenced to solid adamantane at 1.87, D_2O at 4.8, Li_2CO_3 at 0, and $Ce^{17}O_2$ at 877 ppm, respectively. All solution ^1H, ^{13}C, and ^{17}O NMR spectra (Fig. 4) were acquired on an 11.7 T Bruker Avance III HD spectrometer with a single 30º flip angle pulse sequence for ^1H and ^{13}C (10.5 and 9.5 µs, respectively), and an anti-ringing pulse sequence with a 90º-pulse length of 15.5 µs for ^{17}O. The ^{13}C spectra were measured with waltz16 pulse-gated ^1H decoupling. All spectra were measured with a recycle delay of 1, 3, and 0.1 s for ^1H, ^{13}C, and ^{17}O, respectively. The 2D ^1H-^{13}C heteronuclear single quantum correlation (HSQC) experiment was acquired with a recycle delay of 0.8 s, with 256 points on t_1 dimension with 4 transients for each time increment.

Oxygen consumption and gas evolution were measured during cell cycling by monitoring the internal pressure of swagelok cells. Swagelok cells, which were connected to an EL-Cell ECC-Press pressure sensor via Swagelok connections and PEEK tubing. Changes in the internal pressure were recorded. The pressure change was then converted to a change in the number of moles of oxygen consumed or gas evolved during cycling using the ideal gas law. To carry out this conversion it was necessary to determine the internal volume of the swagelok cells. This was carried out using Boyle's law where the volume of the sensor, pressure in the cell and pressure in the sensor were known. The cell and sensor were isolated using a swagelok needle valve. The pressure inside the cell and sensor were adjusted to different values. The needle valve was then opened and the total pressure of the system measured. The following equation based on Boyle's Law was used to determine the cell volume:

$P_cV_c + P_sV_s = P_tV_t$

Where P_c = pressure in cell, V_c = volume of cell, P_s = pressure in sensor, V_s = volume of sensor, P_t = pressure of combined system and V_t = volume of combined system.

This was rearranged to give:

$V_c = (V_s \times (P_t - P_s)) / (P_c - P_t)$

Operando electrochemical mass spectrometry measurements were recorded using a Hiden QGA gas mass spectrometer. Swagelok cells were connected in line with a gas flow system. The gas passing through the cell was sampled by the mass spectrometer. The gas dissolved in the electrolyte was in equilibrium with the flowing gas allowing for the determination of gases evolved during charging. Argon was used as the carrier gas and was flown through the system at 0.8 ml/min, this closely matched the mass spectrometer sampling rate ensuring that almost all the gas evolved during charging was sampled.
Calibration steps were also carried out after cycling to allow for quantitative determination of the gases evolved. Calibration involved pulsing oxygen through the system at a known flow rate for a known period of time. The number of moles of oxygen detected could then be compared the number of moles of oxygen known to have passed through the system. The values obtained during the cycling experiment were then adjusted accordingly.

WILEY-VCH

Supporting Experimental Results and Discussion

1. Characterization of the Ru/SP pristine electrode by SEM, TEM and TGA

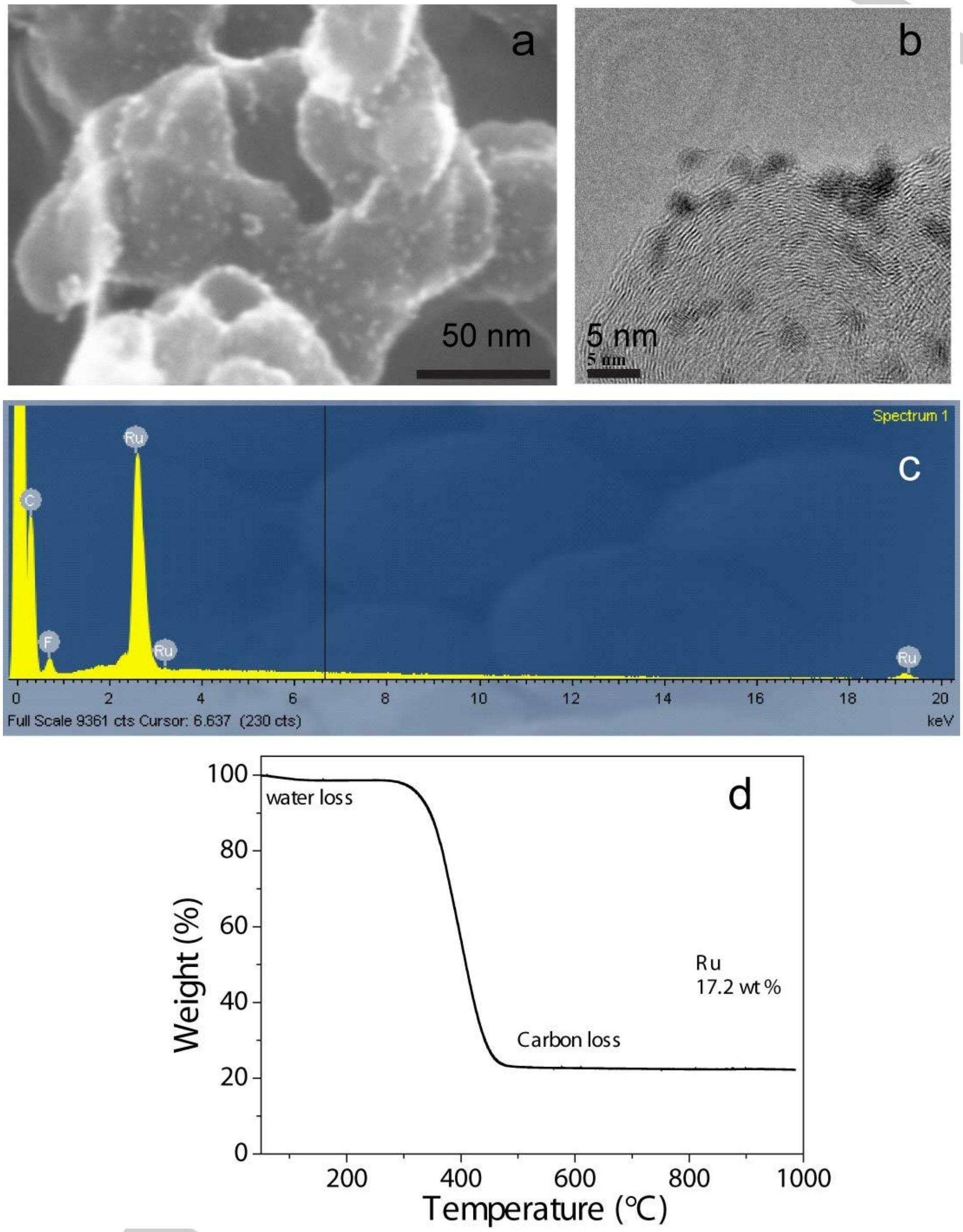

Figure S1. SEM (a), TEM (b) of the synthesized Ru supported on Super P carbon, showing that the Ru catalyst particles are of less than 5 nm and homogeneously dispersed on the carbon; EDX spectrum (c) of the Ru/SP/PTFE electrode confirms the existence of Ru; thermogravimetric analysis (d) shows that the weight loading of Ru is around 17.2 %.

2. Characterizing the discharging process using a nominally dry electrolyte

When a Ru-catalyzed cell is discharged under a nominally dry electrolyte (Figure S2), *operando* pressure measurements (a) show that the discharge process closely follows a two-electron oxygen reduction reaction (e^-/O_2=2.1), supporting Li_2O_2 formation; the corresponding SEM measurement (b) revealed a dominant toroidal morphology that is characteristic for Li_2O_2. XRD of the sample (c) further verify that Li_2O_2 was the main crystalline phase identified, although LiOH and other side reaction products (lithium acetate, formate and methoxide, Figure 2E in main text) were also found; these side reaction products were also observed by other groups for a Li_2O_2 chemistry using a nominally dry DMSO-based electrolyte (references [2]e-f).

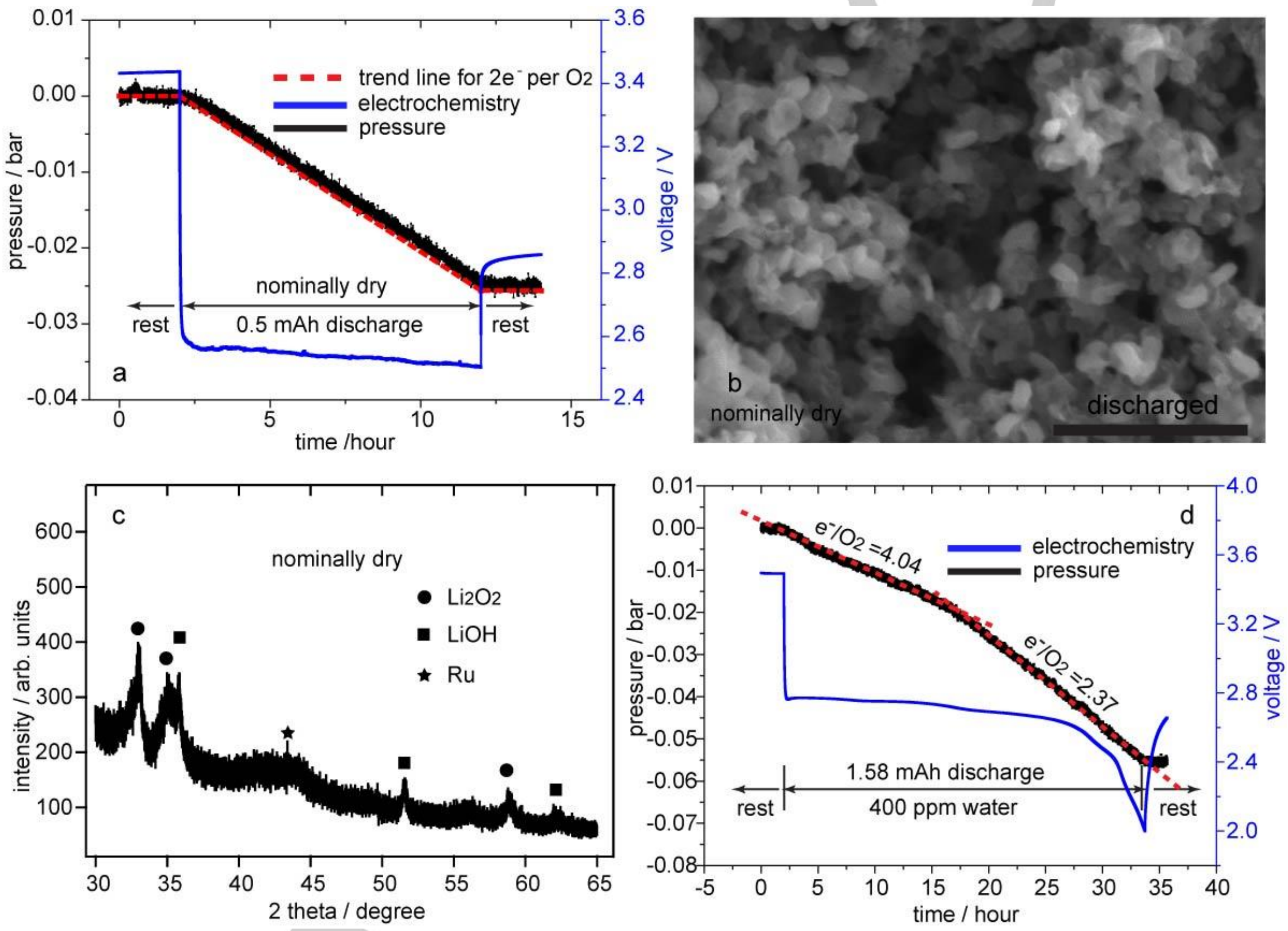

Figure S2. *Operando* electrochemical pressure measurements of discharging Ru-catalyzed batteries using a nominally dry electrolyte (a) and that with 400 ppm added water; SEM (b) and XRD (c) characterization of the discharged electrodes using a nominally dry 1M LiTFSI/DMSO electrolyte. The scale bar in (b) represent 2 microns. The small toroidal particles are of 100-200 nm in size in a fully discharged electrode (1.7 mAh), which is consistent with the low intensity of any reflections from crystalline phases in the XRD pattern and the poor solvating properties the electrolyte in the absence of water.

When a Ru-catalyzed cell is discharged in an DMSO electrolyte with 400 ppm water (Figure S2d), approximately half of the (initial) discharge process follows a four-electron oxygen reduction reaction and the subsequent discharge seems to be closer to a two-electron oxygen reduction process (e^-/O_2=2.37); this

transition is probably because the LiOH formation in the initial discharge process consumes water ($O_2 + 4e^- + 4Li^+ + 2H_2O \rightarrow 4LiOH$) and when the residual water content becomes low, the discharge then starts to operate via the Li_2O_2 chemistry ($O_2 + 2e^- + 2Li^+ \rightarrow Li_2O_2$) or a mixture of the two chemistries. It is thus clear that the water content in the DMSO electrolyte plays a decisive role in determining the chemistry in the Ru-catalyzed battery: at nominally dry conditions (within tens of ppm water) the battery operates via two-electron Li_2O_2 formation; at intermediate water contents (hundreds of ppm) the battery can operate via formation of a Li_2O_2 and LiOH mixture depending on the depth of discharge; at even higher water contents (beyond thousands of ppm) the cell operates entirely via LiOH formation.

3. Electrochemistry of the Ru-catalyzed battery with higher water contents (> 5%)

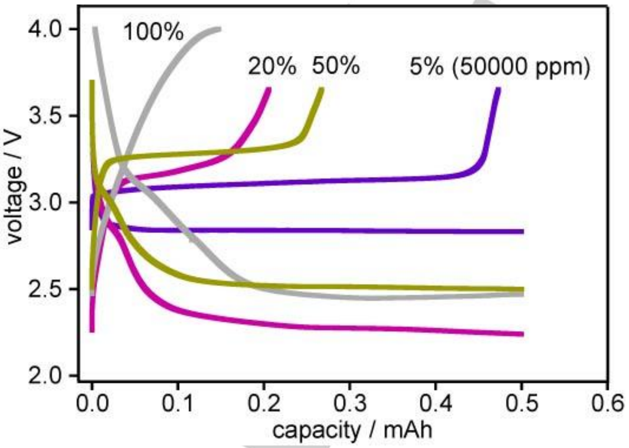

Figure S3. Electrochemical profiles of Li-O_2 batteries using a Ru/SP electrode and different electrolytes, including 50,000, 200,000, 500,000 ppm (50%) H_2O in 1M LiTFSI/DMSO, and 1 M LiTFSI/H_2O. LFP was used as the counter electrode for all cases. As the water content increases beyond 50,000 ppm, the discharge and charge voltage gaps also widen.

4. ^{1}H and ^{7}Li solid state NMR characterization of the discharged electrodes with other metal catalysts

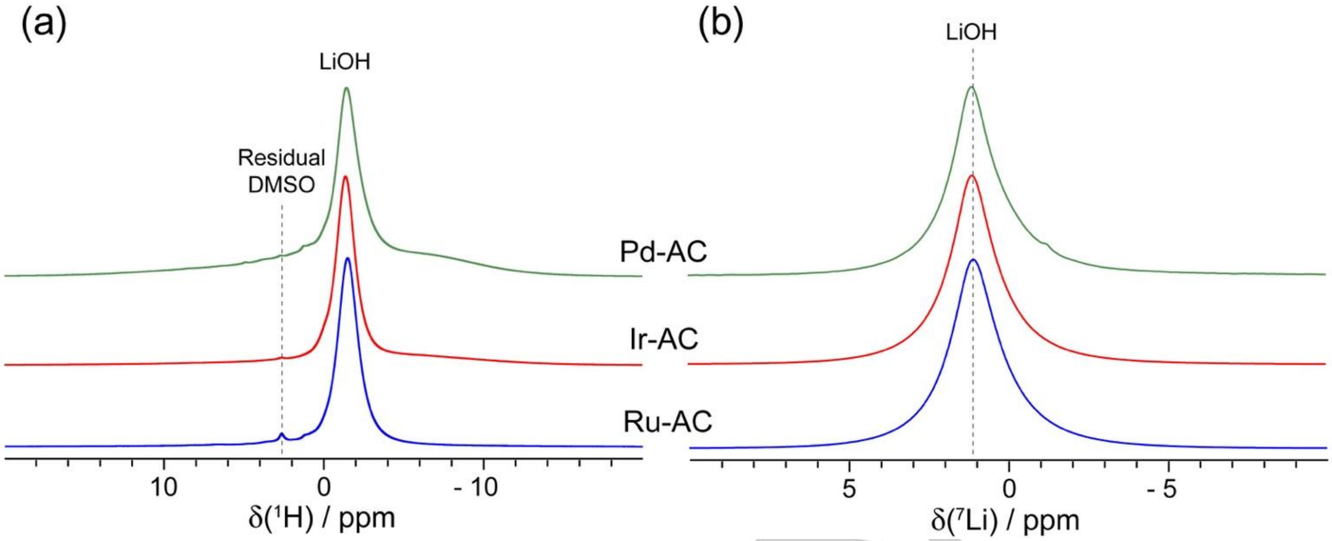

Figure S4. ^{1}H (a) and ^{7}Li (b) solid state NMR spectra of Ru-activated carbon (AC), Ir-AC, Pd-AC electrodes after being discharged in Li-O_2 cells using 1 M LiTFSI/DMSO electrolyte with 4000 ppm water. A single resonance at -1.5 ppm in the ^{1}H spectrum and one at 1.1 ppm in the ^{7}Li spectrum suggest LiOH is the dominant discharge product for Pd, Ir, Ru catalysts.

WILEY-VCH

5. The role of Ru on the catalysis of LiOH formation

The reaction, $2Li_2O_2 + 2H_2O \rightarrow 4LiOH + O_2$, is thermodynamically favorable, with a standard free energy change of -149.26 kJ/mol. When Li_2O_2 is in contact with H_2O, all the Li_2O_2 will spontaneously convert to LiOH. The question is, how fast does this conversion occur?

To clarify the roles of Ru in the battery discharge process, we have conducted a comparison between the two cases: (1) a SP electrode discharged using a 1M LiTFSI/DMSO electrolyte with 4000 ppm added water; (2) a Ru/SP electrode discharged using the same electrolyte as (1). Their corresponding XRD patterns are summarized below in Figure S5a.The discharged SP electrode contained mainly Li_2O_2 (a), although reflections due to LiOH were also identified. The corresponding SEM images (b) revealed a dominant toroidal morphology supporting Li_2O_2 formation, but large crystals with a flower-like morphology were also observed (highlighted by an arrow in b), morphologies that are generally observed for LiOH. *Operando* pressure measurements (c) for case (1) shows that the discharge follows an e^-/O_2 molar ratio of 2.2, progressively deviating from a process of 2 electrons per reduced O_2, which could be due to the water slowly reacting with Li_2O_2 to form LiOH, consistent with a minor process involving LiOH formation, and other side reactions. By contrast, for the Ru/SP electrode discharged in the same electrolyte, only LiOH was identified, the discharge following an e^-/O_2 molar ratio of 4 in the same electrolyte: only LiOH was identified, the discharge following an e^-/O_2 molar ratio of 4 in throughout whole course of discharge (Figure 1 and 2 in main text). The above observations support that the LiOH formation is effectively catalyzed by Ru (and other metal catalysts in Figure S4), without which two-electron Li_2O_2 formation is still the dominant discharge process, even in the presence of water.

A pre-discharged a Ru/SP in a dry LiTFSI/DMSO electrolyte was then exposed to an electrolyte containing 4,000 ppm water. The corresponding XRD patterns (Figure S5a) show that the discharged Ru/SP electrode in a dry electrolyte contained mainly Li_2O_2 (also evidenced in Figure S2); after soaking the pre-discharged electrode in the electrolyte with 4000 ppm water for 10 hours (same as a galvanostatic discharge period for the SP battery), all the Li_2O_2 reflections disappeared and the LiOH reflections became dominant and intense, whereas exposing the Li_2O_2 product on SP electrode to a 4000 ppm water-added electrolyte for 10 hours did not lead to dominant LiOH formation. This contrast in converting Li_2O_2 to LiOH indicates that Ru chemically catalyzes the reaction, $2Li_2O_2 + 2H_2O \rightarrow 4LiOH + O_2$. It is probable that the electrochemical formation of LiOH in the current Ru/SP system proceeds via first Li_2O_2 generation ($O_2 + 2e^- + 2Li^+ \rightarrow Li_2O_2$) and then chemical reaction of Li_2O_2 with H_2O to eventually form LiOH ($2Li_2O_2 + 2H_2O \rightarrow 4LiOH + O_2$); overall, however, the reaction is $O_2 + 4e^- + 4Li^+ + 2H_2O \rightarrow 4LiOH$. Nonetheless, another route via Ru-catalyzing the reaction of H_2O with LiO_2 to form LiOH is also possible ($O_2 + e^- + Li^+ \rightarrow LiO_2$, $4LiO_2 + 2H_2O \rightarrow 4LiOH + 3O_2$); overall it is also a four-electron oxygen reduction reaction.

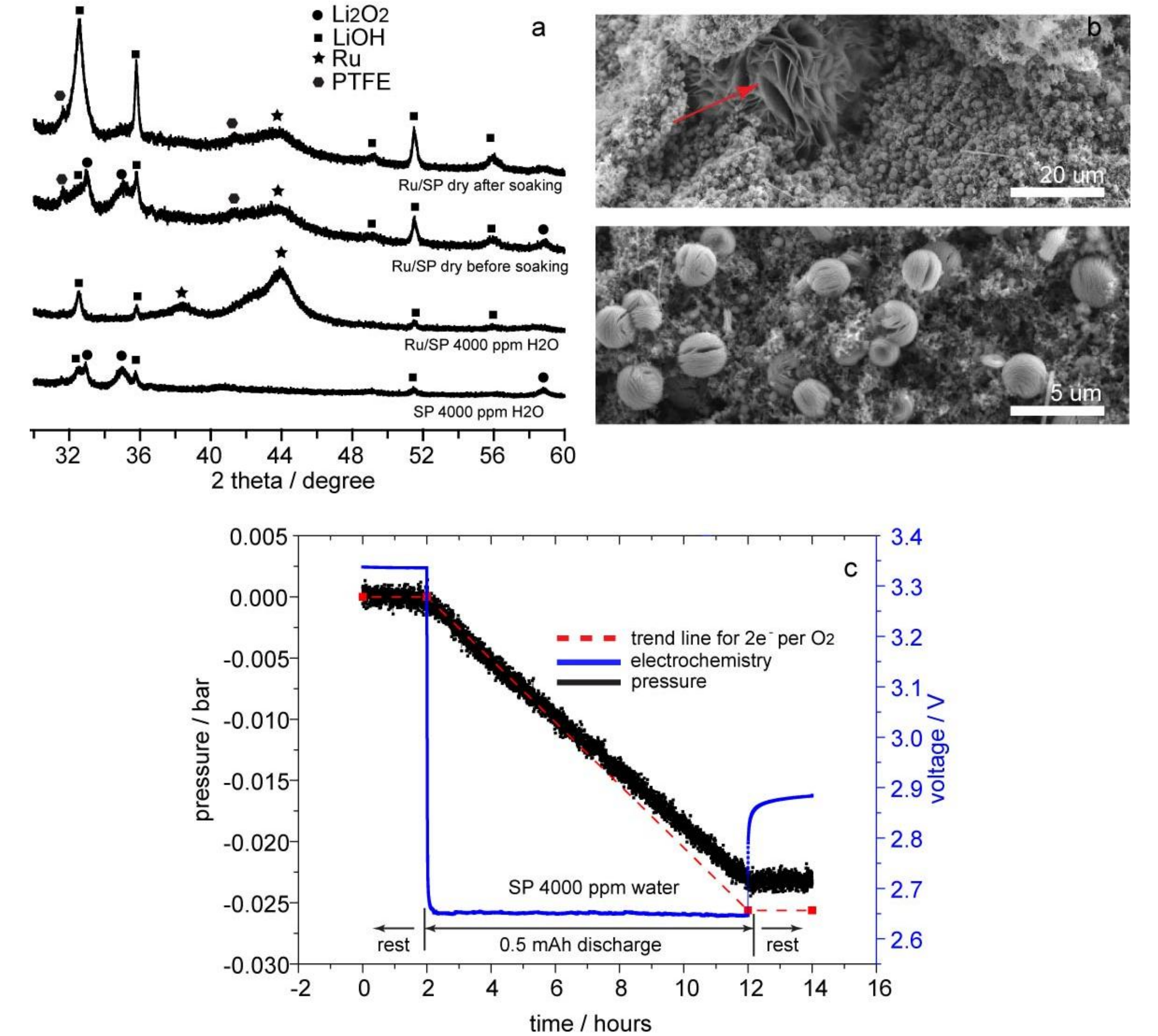

Figure S5. XRD measurements (a) of discharged SP and Ru/SP electrodes (bottom two patterns) using a 1 M LiTFSI/DMSO electrolyte with 4000 ppm water, and a pre-discharged Ru/SP electrodes using a nominally dry electrolyte before and after soaking it in an electrolyte with 4000 ppm added water for 10 hours; SEM characterization (b) of a discharged SP electrode using a 1 M LiTFSI/DMSO electrolyte with 4000 ppm water and the corresponding *operando* electrochemical pressure measurement for the discharging process.

6. ^{1}H and ^{13}C solution NMR characterization of the electrolyte after discharge, charge and soaking with LiOH under O_2

The residual electrolytes (1 M LiTFSI/DMSO with 45,000 ppm water) at the end of a full discharge, charge and an electrolyte that had been soaking LiOH powder under O_2 for 30 days were evaluated using solution NMR. Both ^{1}H and ^{13}C solution NMR measurements (Figure S6) show that negligible side reactions occur during electrochemical LiOH formation on discharging or LiOH soaking, again suggesting that the LiOH formation process involves few side reactions and LiOH itself is chemically stable in DMSO. On charging, however, considerable dimethyl sulfone forms (further discussion on Figure 4, main text).

WILEY-VCH

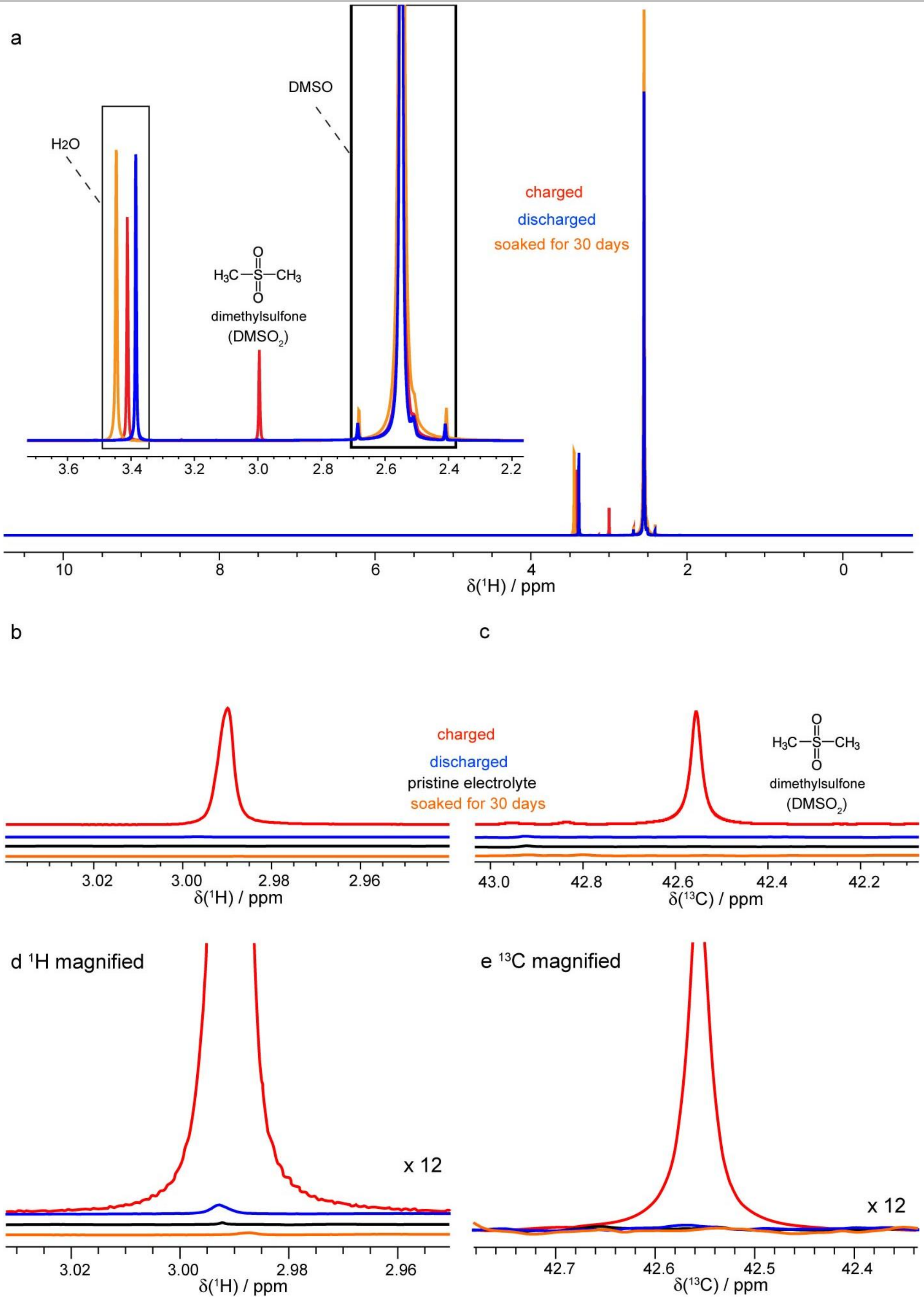

Figure S6. ^{1}H (a, b, d) and ^{13}C (c, e) solution NMR measurements of 1 M LiTFSI/DMSO (45,000 ppm H_2O) electrolytes after various treatments. (a) compares the electrolytes after the first discharge (1 mAh), after 3rd charge, and the DMSO solvent soaked with LiOH under an O_2 atmosphere for 30 days, the inset showing an enlarged image for the 2.2-3.6 ppm region. (b-e) focuses on the $DMSO_2$ region in the corresponding ^{1}H and ^{13}C spectra of the aforementioned samples, and shows that neither electrochemical LiOH formation on discharging nor soaking DMSO with LiOH under O_2 led to any significant $DMSO_2$ formation, but electrochemically charging the battery caused considerable $DMSO_2$ accumulation in the electrolyte.

7. Long-term cycling of the Ru-catalyzed Li-O_2 battery under conditions of LiOH formation

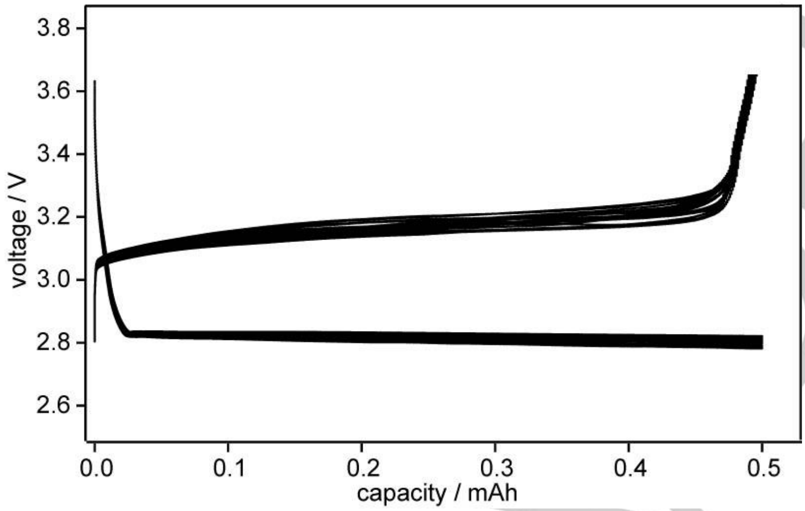

Figure S7. Long term electrochemical cycling (50 cycles) of a Li-O_2 battery using a Ru/SP electrode and 1 M LiTFSI/DMSO electrolyte with 50,000 ppm added H_2O. Typically, about 0.1-0.15 ml electrolyte is used in a single cell. Based on the reaction scheme: (2) $4LiOH \rightarrow 4Li^+ + 4e^- + 4^{\cdot}OH$ (hydroxyl radicals); (3) DMSO + $2^{\cdot}OH \rightarrow DMSO_2 + H_2O$. One DMSO molecules will be oxidized to a $DMSO_2$ per 2 electrons. For a battery that has been cycled 100 times with a capacity of 0.5 mAh per cycle, 0.066 ml DMSO will have been consumed; this is still less than the total amount of electrolyte used. The amount of DMSO in a cell can thus sustain the charge reaction for many cycles.

8. Further discussion on the LiOH oxidation potential with different water contents

The thermodynamic potential of LiOH oxidation reaction on charging, $4LiOH \rightarrow 4Li^+ + 4e^- + 2H_2O + O_2$, is dependent on the chemical potential or the activity of water in the electrolyte. The standard thermodynamic potential for the above reaction is $E^o=\Delta G^o/-nF=3.32$ V. If we approximate the activity of water in the electrolyte to the water content, one derives equilibrium voltages of 3.2 V at 100 ppm, 3.25 V at 4000 ppm and 3.28 V at 50,000 ppm water contents. The value obtained at 50,000 ppm water is very close to the value at standard conditions, suggesting that the properties of the water molecules within the electrolyte (e.g., chemical environment in the solution) are close to those of bulk water. To test this hypothesis, ^1H solution NMR measurements were conducted on the electrolyte with a varying water content: 10 ppm, 400

ppm, 4000 ppm, 50,000 ppm, 500,000 ppm and bulk water (95%), as shown in Figure S8. It is clear, however, that at 50,000 ppm water its chemical shift is closer to that at 10-400 ppm, rather than being similar to that of bulk water. Therefore, approximating the water activity by simply using its concentration within the electrolyte is not an accurate method for deriving the equilibrium voltage and may provide an overestimate of the voltage. More careful evaluation of the enthalpy and entropy change of the water in non-aqueous electrolytes is needed to derive the chemical potential change of water and more accurate estimates for the charging potentials. The very different water environments even at 5 wt% water may be related to the salts in the electrolye (along with the DMSO).

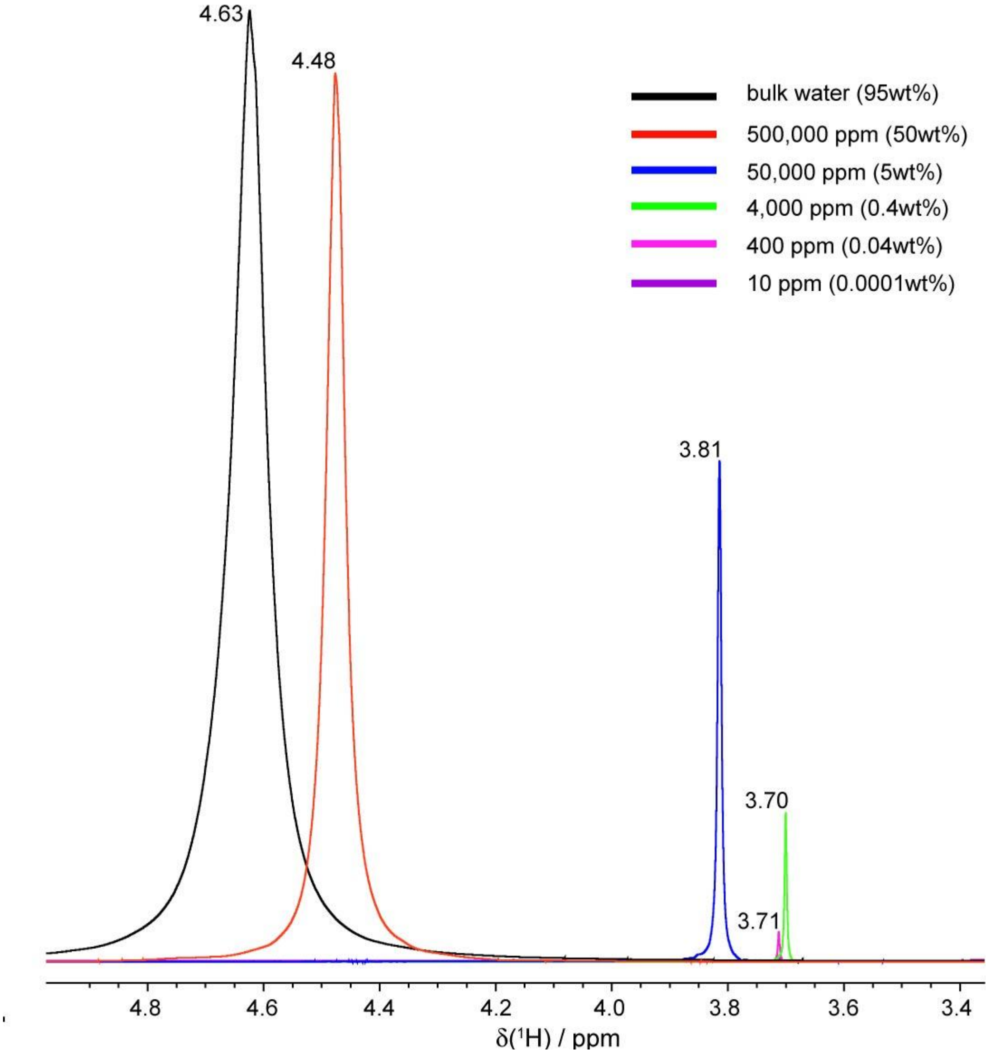

Figure S8. ^{1}H solution NMR measurements of 1 M LiTFSI/DMSO electrolyte with different water contents: 10 ppm, 400 ppm, 4000 ppm, 50000 ppm, 500000 ppm and bulk water. 5% d6-DMSO was used added so as to shim the magnet, deuterated DMSO being used so as to mimic the solvation conditions of water in the actual (non-deuterated) electrolyte. In general, as the water level increases, the ^{1}H resonance of water shifts closer towards the bulk value (4.8 ppm). At 10, 400 and 4000 ppm water contents, the ^{1}H resonances are observed at around 3.71 ppm. Further increasing the water content by an order of magnitude to 50,000 ppm, the resonance shifts by 0.1 ppm. Increasing the water content by another order of magnitude to 500,000 ppm (50%) water, a large positive shift towards bulk water by ~0.67 was seen.

We suggest (and provide supporting experimental evidence) that the battery charges via a different mechanism: the first step is an electrochemical one, where LiOH is decomposed to form Li^+, e^- and ·OH radicals; the second step is a chemical one, where ·OH radicals react with DMSO to form of $DMSO_2$. The chemical reaction of DMSO with ·OH radicals is kinetically fast, and therefore the rate limiting step in the charging process would be: $LiOH \rightarrow Li^+ + e^- + \cdot OH$, also the first elementary step toward LiOH decomposition to evolve O_2, which is found to take place at voltages close to 3.1V in wet electrolytes. The electrochemical reaction in a cell sets the measured voltage of the cell, not the overall ΔG^o of the reaction which may also involve a subsequent chemical reaction. The subsequent chemical reaction serves to drive the equilibrium towards the formation of $DMSO_2$.

From the discussion in Figure S2 we conclude that at nominally dry conditions and at 400 ppm of water, the battery can operate via entirely Li_2O_2 chemistry or a mixture of LiOH and Li_2O_2 formation, respectively. The large overpotential drop (~0.4 V in Figure 1A) on charging from 0 ppm to 50,000 ppm water can be largely attributed to the transition from charging predominantly Li_2O_2 (0 ppm), Li_2O_2 and LiOH mixtures (100, 250 ppm), and purely LiOH (>4000 ppm), rather than a result of equilibrium voltage shift due to water contents.

The change in reaction kinetics upon addition of water in the electrolyte can be explained by considering that the rate limiting step is the formation of ·OH radicals, as proposed above. The reaction will proceed via dissolution of LiOH forming soluble Li^+ and OH^- ions, and the latter is then oxidized to form ·OH radicals in the first (electrochemical) step. It is reasonable to propose that at beyond 4000 ppm, further increasing the water content will more efficiently solubilize LiOH and improve the kinetics of removing LiOH, further decreasing in the voltage gap from 4000 ppm to 50,000 ppm water.

To conclude, the changes in overpotential are first due to a change in mechanism from Li_2O_2 to LiOH chemistry and then to improvements in kinetics due to enhanced LiOH solubility.

Author Contributions

T. Liu and C.P. Grey conceived and designed the project. T. Liu and Z. Liu performed the electrochemistry, SEM and XRD measurements. T. Liu, Z. Liu and G. Kim performed the NMR measurements. T. Liu and J.T. Frith performed the *operando* electrochemical pressure and mass spectrometry measurements. T. Liu wrote the original draft and all authors discussed the science and commented on the paper. N. Garcia-Araez and C.P. Grey supervised the project.

WILEY
